# Retraction: A Novel Herbal Medicine, KIOM-C, Induces Autophagic and Apoptotic Cell Death Mediated by Activation of JNK and Reactive Oxygen Species in HT1080 Human Fibrosarcoma Cells

**DOI:** 10.1371/journal.pone.0355655

**Published:** 2026-08-13

**Authors:** The PLOS One Editors

Following the publication of this article [1], concerns were raised regarding results presented in Figs 6, S2, and S3, the statistical methodology, and undeclared competing interests. Specifically,

- The following panels appear to fully or partially overlap, despite representing different experimental conditions:
  - Fig 6B panel c, Fig 6D JNK siRNA KIOM-C (500 µg/ml) +, and the third Fig S2B panel
  - Fig S2A p-ERK and Fig S3D AGS p-ERK panels
  - The first and the fourth S3E AGS panels
- The methodology section only reports the use of Student’s t-test, and does not explain how the analysis was adjusted for multivariate comparisons or corrected for multiple pairwise comparisons.
- The Competing interests statement of this article states that the authors have declared that no competing interests exist, despite the corresponding author being listed as an inventor on patent PCT/KR2010/000107 [2] which is linked to the novel herbal medicine KIOM-C tested in this study.

Regarding the apparent panel overlap concerns, the first author stated that, in the absence of the original image files, they could not determine conclusively how the apparent overlaps arose or exclude the possibility of inadvertent figure assembly errors. In the absence of the original image files, these concerns cannot be fully resolved.

Regarding the statistical methodology concerns, the first author clarified that no multivariate analysis was performed and that no adjustment for multiple comparisons was applied. Statistical differences were assessed between the relevant control and treatment groups. PLOS remains concerned about the methodological approach used for the data analysis reported in this study.

Regarding the undisclosed competing interest, the first author stated that, at the time of publication, the authors did not fully appreciate that the corresponding author’s inventorship on a KIOM-C-related patent application should have been disclosed as a competing interest.

In light of the unresolved concerns, which call into question the reliability and integrity of the published results, the *PLOS One* Editors retract this article.

AK and NHY did not agree with the retraction. MI,TK, and JYM either did not respond directly or could not be reached.
